# Discordance Rate of HER2 Status in Primary Gastric Carcinomas and Synchronous Lymph Node Metastases: A Multicenter Retrospective Analysis

**DOI:** 10.3390/ijms151222331

**Published:** 2014-12-03

**Authors:** Antonio Ieni, Valeria Barresi, Rosario Caltabiano, Alessia Caleo, Luca Reggiani Bonetti, Salvatore Lanzafame, Pio Zeppa, Rosario Alberto Caruso, Giovanni Tuccari

**Affiliations:** 1 Department of Human Pathology “Gaetano Barresi”, Section of Anatomic Pathology, University of Messina, Messina 98123, Italy; E-Mails: calaienco@hotmail.com (A.I.); vbarresi@unime.it (V.B.); rosariocaruso@tin.it (R.A.C.); 2 Department “G.F. Ingrassia”, Section of Anatomic Pathology, University of Catania, Catania 95123, Italy; E-Mails: rosario.caltabiano@unict.it (R.C.); lanzafas@unict.it (S.L.); 3 Department of Medicine and Surgery, University of Salerno, Salerno 84131, Italy; E-Mails: alessia.caleo@unisa.it (A.C.); pzeppa@unisa.it (P.Z.); 4 Department of Diagnostic Medicine, Clinics and Public Health, Section of Pathologic Anatomy, University of Modena, Modena 41121, Italy; E-Mail: reggiani.luca@policlinico.mo.it

**Keywords:** human epidermal growth factor receptor 2 (HER2), gastric cancer, synchronous lymph nodes, metastases, prognosis

## Abstract

Background: The assessment of human epidermal growth factor receptor 2 (HER2) gene amplification is essential in order to identify those patients affected by advanced gastric cancer who may benefit from Trastuzumab targeted therapy. Materials and Methods: With the aim to investigate the concordance rate in HER2 status between primary gastric carcinoma (GC) and synchronous lymphnode metastases, we investigated HER2 status in a cohort of 108 surgical formalin-fixed paraffin-embedded specimens of GC and matched synchronous metastatic lymph nodes collected from three different units of Anatomic Pathology in southern of Italy. Fleiss-Cohen weighted k statistics were used to assess the concordance rate of HER2 status. Results: HER2 amplification was observed in 17% of primary GCs and the overall concordance rate with corresponding nodal metastases was 90.74%. Changes in HER2 status between primary GC and matched synchronous metastases were evidenced in 10 (9.26%) cases. Of these, 6 cases were HER2 amplified in the primary GC and not amplified in the metastases, while 4 were HER2 not amplified in the primary tumour and amplified in the lymph node metastases. Conclusions: Although at present the simultaneous determination of HER2 in advanced gastric cancer and corresponding metastatic lymph nodes is not mandatory, the possibility that the synchronous metastases of GC have a different HER2 status from that of the primary tumour is of remarkable significance; Indeed this may have influence on the therapeutic management and prognosis of the patients.

## 1. Introduction

Recent studies showed that the gene expression profile of breast and colorectal cancer may display significant variation among paired samples of primary tumours, disseminated neoplastic cells and patient metastases [1,2,3,4,5]. In particular, a significant discordance rate in human epidermal growth factor receptor 2 (HER2) receptor status was retrospectively demonstrated between primary and recurrent/metastatic breast cancer [6,7,8,9,10]. This discrepancy may depend upon changes in HER2 receptor expression during the natural history of the tumour and has clinical and therapeutic relevance [11]. Of note, discordant cases may be characterized by either positive or negative conversion in HER2 status in metastatic asynchronous or synchronous axillary lymph nodes compared to the primary breast carcinoma [12,13,14,15,16].

HER2 amplification may be also observed in gastric carcinomas (GC), with a prevalence ranging between 7.7% and 25%. The frequency of HER2 amplification in GC varies according to the localization and histotype of the tumour [17,18,19,20]. Indeed higher rates of amplification were shown in unusual aggressive histotypes, such as the hepatoid variant [21,22]. At present, only a few studies investigated the heterogeneity in HER2 status in paired samples of primary and metastatic GC [19,23,24,25] and evidenced a low rate of discordance in HER2 amplification with either positive or negative conversion [19,24,25]. Thus, the aim of this study was to assess the concordance in HER2 status between primary GC and synchronous metastatic lymph nodes. The analysis was carried out on paired samples collected during the same surgical procedure and submitted to same fixation methodology in order to avoid any technical bias depending on external factors and to give insight into the biological reasons underlying changes in HER2 status.

## 2. Results

The histopathological and immunohistochemical characteristics of the cases included in the study are summarized in Table 1. Tumour localization in the stomach was: Lower third in 52 cases, middle third in 38 and upper-third in 18, 7 of which were located at the gastro-oesophageal junction. Histopathological diagnosis according to the World Health Organization (WHO) criteria was: Adenocarcinoma (tubular, papillary, tubulo-papillary and mucinous) in 62 cases, poorly cohesive carcinoma in 35 and mixed in 11. According to Lauren’s Classification 62 cases were recorded as intestinal type, 35 as diffuse and 11 cases as mixed. Fifty-five out of the 108 GC were classified as low-grade and 53 as high-grade tumours. With reference to the pTNM staging, 1 case was at stage I, 39 at stage II, 53 stage at III and 15 at stage IV. T1 and T2 as well as T3 and T4 cases were grouped for the statistical analyses. Similarly, N2 and N3 cases were considered altogether and compared to N1 tumours.

**Table 1:** Clinico-pathological parameters in relation to HER2 status in 108 primary gastric carcinoma (GC) cases.

Parameter	Number	HER2		*p*
		Amplified	Not Amplified	
**Gender**				
Male	67	13	54	0.4781 (NS)
Female	41	5	36	
**Site**				
Lower	52	10	42	0.7471 (NS)
Middle	38	5	33	
Upper	18	3	15	
**Lauren Histotype**				
Intestinal	62	15	47	0.0496
Diffuse	35	2	33	
Mixed	11	1	10	
**WHO histotype**				
Tubular	62	15	47	0.0496
Poorly cohesive	35	2	33	
Mixed	11	1	10	
**Grade**				
Low	55	10	45	0.8633 (NS)
High	53	8	45	
**Stage**				
I–II	40	7	33	0.929 (NS)
III–IV	68	11	57	
**T**				
1–2	15	5	10	0.1354 (NS)
3–4	93	13	80	
**N**				
1	39	5	34	0.5909 (NS)
2–3	69	13	56	

GC: Gastric carcinoma; NS: Not significant; T: Tumour; and N: Node.

With regards to HER2 immunohistochemical assessment, 18 (17%) primary GC were scored as 3+, 7 (6.5%) as 2+, 8 (7.5%) as 1+ and 75 (69%) as 0 (no staining). Fluorescence *in situ* hybridization (FISH) analysis revealed no amplification in all of the cases scored as 2+ and confirmed the amplification of all cases with a 3+ score. Thus, on the whole, among primary GC, HER2 was considered as amplified in 18 cases (17%) and not amplified in a total of 90 cases (83%).

When the synchronous nodal metastases were considered, HER2 was scored as 3+ in 16 (15%) cases, as 2+ in 4 (4%), as 1+ in 5 (5%) cases and as 0 in 82 (76%) cases. When FISH analysis was carried out in the 2+ tumours, no gene amplification was found.

The *k* value for the concordance rate in the HER2 status between primitive tumours and metastases was 0.651 (substantial agreement).

**Figure 1:**
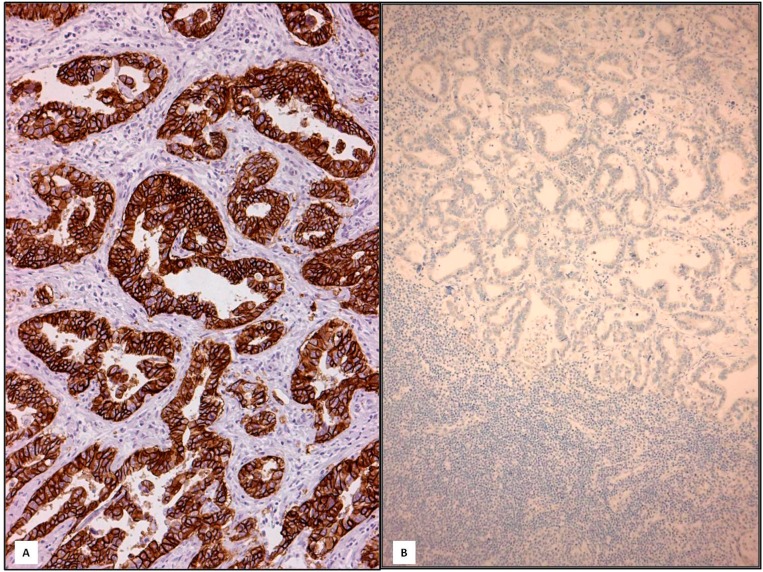
In intestinal type primary GC, the 3+ score present in neoplastic glands (**A**, 200×) disappeared in metastatic lymph node (negative conversion) (**B**, 160×) (Immunohistochemistry, Mayer’s haemalum counterstain).

**Figure 2:**
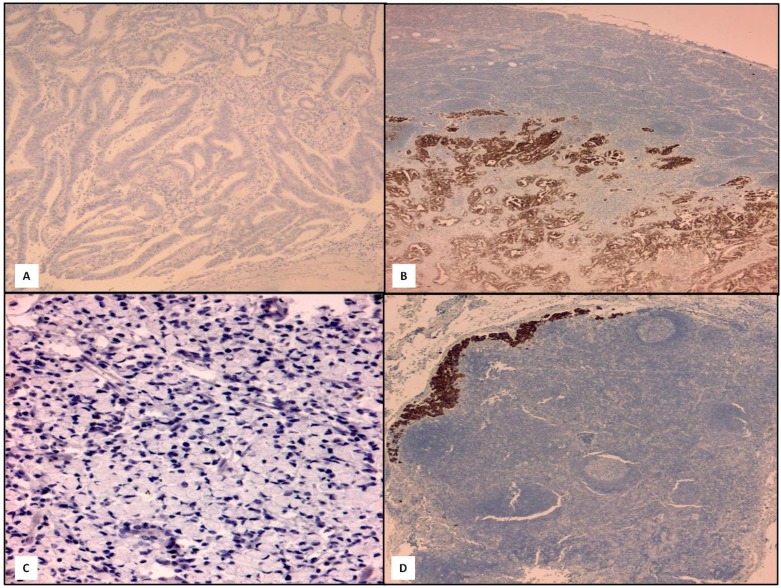
Two cases of positive conversion, one example in the GC intestinal type (**A**, 160×; **B**, 120×) and one in diffuse type (**C**, 160×; **D**, 120×) (IHC, Mayer’s haemalum counterstain).

The overall concordance rate was 90.74%; In detail, 86 cases were concordantly not amplified for HER2 in primary GC and corresponding nodal metastases, while 12 cases were HER2 amplified in both primitive and metastatic tumours (Table 2). Changes in HER2 status between primary GC and matched synchronous metastases were evidenced in 10 (9.26%) cases, the characteristics of which are summarized in Table 3. In detail, 6 cases were HER2 amplified in the primary GC and not amplified in the metastases (negative conversion) (Figure 1), while 4 of the discordant cases were HER2 not amplified in the primitive tumour and amplified in the lymph node metastases (positive conversion) (Table 3) (Figure 2).

No significant differences in the various clinico-pathological parameters were evidenced between discordant and concordant GC (Table 2). On the other hand, HER2 amplification was significantly more frequent in the intestinal-type GC compared to diffuse-type (*p* = 0.0496) (Table 1).

**Table 2:** Clinico-pathological parameters in relation to concordance of HER2 status.

Parameter	Discordant GC	Concordant GC	*p*
**Gender**			
Male	9	58	0.1162 (NS)
Female	1	40	
**Site**			
Lower	5	47	0.1281 (NS)
Middle	1	34	
Upper	4	17	
**Lauren Histotype**			
Intestinal	5	57	0.5578 (NS)
Diffuse	3	32	
Mixed	2	9	
**WHO histotype**			
Tubular	5	57	0.5578 (NS)
Poorly cohesive	3	32	
Mixed	2	9	
**Grade**			
Low	6	49	0.7867 (NS)
High	4	49	
**Stage**			
I–II	4	36	0.8886 (NS)
III–IV	6	62	
**T**			
1–2	3	12	0.2862 (NS)
3–4	7	86	
**N**			
1	4	35	0.9388 (NS)
2–3	6	63	

GC: Gastric carcinoma; NS: Not significant; T: Tumour; and N: Node.

**Table 3:** Ten cases with changes in HER2 status between the primary GC and corresponding synchronous nodal metastases.

Sex	Stage	pT	pN	Histotype	Grade	Primary GC	Metastatic LN
M	III	3	3	Mixed	High	3+	0
F	III	2	3	Intestinal	Low	3+	0
M	II	2	1	Intestinal	Low	3+	1+
M	III	2	3	Intestinal	Low	3+	0
M	II	3	1	Mixed	Low	3+	0
M	III	3	3	Intestinal	High	3+	0
M	II	3	1	Mixed	Low	0	3+
M	II	3	1	Diffuse	Low	2+ *	3+
F	III	3	3	Intestinal	High	0	3+
F	IV	3	3	Diffuse	High	0	3+

GC: Gastric carcinoma; pT: Post-surgery tumour; pN: Post-surgery node; LN: Lymph nodes; and * Not amplified.

## 3. Discussion

The importance of standardization in the assessment of HER2 status in GC has been underlined by a consortium of expert pathologists in European countries [18,26,27]. Indeed HER2 status has been identified as a significant additional prognostic parameter in advanced GC [28]. Interestingly, the possible discordance in HER2 status between primary tumour and matched metastases has been shown in both breast and stomach cancers [7,9,10,11,15,19,24,25,29,30]. As a consequence, the need to assess HER2 status not only in the primary tumour, but also in its metastases, was recently emphasized. However, studies investigating the changes in HER2 status mainly considered recurrent, loco-regional or metachronous lesions, while modification in HER2 status between paired primary neoplasm and synchronous nodal metastases was explored in only a few analyses [15,23,25].

In the present study we retrospectively analyzed HER2 expression in whole sections of surgical specimens of GC and matched metastatic lymph nodes. All of the samples—Primary and metastatic tumours—Had been collected during the same surgical procedure and consequently submitted to the same fixation methodology. By doing so, we could avoid technical and therapy-related variability.

The results of our analysis can be summarized as follows. Firstly, we could confirm the presence of a high level of concordance in HER2 status between primary GC and corresponding lymph node metastases (90.74%), as it was previously shown in studies on metachronous metastases (87.5%–94.9%) [19,23,24]. In our cohort of cases, all the amplified cases were scored as 3+ by immunohistochemistry and the rate of HER2 amplification was 17%, similarly to the HER2 positivity rate (19.2%) reported in the literature for GC [26].

Our second relevant finding was that HER2 status differed between primary GC and its nodal metastases in 9.26% of cases, *i.e.*, in 10 out of the 108 analyzed GC. In detail, a negative conversion was observed in 6 cases, which were HER2 amplified in the primitive carcinomas and negative in the metastases, while a positive conversion was found in 4 cases, which were HER2 negative in the GC and changed to positive in the lymph node metastases. Of note, a similar discordance rate in HER2 status (9.8%) was recently reported in a series of 102 GC and paired metastatic lymph nodes in which a negative conversion in lymph nodes was observed in 4 cases, while a positive conversion in lymph nodes was found in 6 cases [25].

Change in HER2 status between primary GC and synchronous lymph node metastases may have relevant clinical impact. Indeed, at present patients with advanced GC are candidates for therapy with Trastuzumab on the basis of HER2 positivity in the GC only, while our findings suggest that HER2 status should be assessed, not only in metachronous metastases, but also in synchronous nodal metastases before treatment decision. As a matter of fact, testing HER2 expression in only the primary GC may exclude from the targeted treatment a percentage of patients with a negative primary tumour but positive metastases. Besides, similar considerations were already underlined in breast cancer, in which positive and negative conversion of HER2 status in lymph node metastases compared to the primary tumour has been documented [31].

Intratumoural heterogeneity in HER2 status in different areas of the primary lesion seems to be more common in gastric than in breast cancer [26]. On this evidence, biopsies or tissue microarray assays (TMA) cannot be considered as adequate specimens for the assessment of HER2 expression. On the other hand, the analysis of whole sections obtained from two significant areas of primary GC as well as from at least four metastatic lymph node tissue blocks may minimize technical sampling errors as a cause of HER2 discordance between primary and metastatic GC. Hence, we may hypothesize that HER2 discordance in our cases may be related to the biological tendency of the tumour leading to the selection of a new neoplastic cell clone. It is tempting to speculate that HER2 positive cells may appear as sub-clones in metastatic lymph nodes as a result of disease progression (positive conversion). Besides, loss of HER2 amplification (negative conversion) in metastatic deposits could not be merely attributed to the development of resistance to Trastuzumab therapy, since our patients had not been subjected to any neo-adjuvant treatment.

## 4. Materials and Methods

One hundred and eight surgical specimens of GC (67 male and 41 female patients; Mean age of the patients: 68.06 years; Range 39–95 years) and corresponding synchronous lymph node metastases were retrospectively collected from three different units of Anatomic Pathology in southern Italy. None of the patients had received neo-adjuvant chemotherapy or other therapies for their tumour.

Data on the site, histotype according to the WHO 2010 or Lauren’s classifications as well as on the HER2 status of the tumour were available in all the cases. The patients’ personal details were non-identifiable and all the patients had provided written consent to their medical information being used for research purposes, according with the Helsinki declaration. Approvals for the retrospective study were obtained from the Local Ethics Committees of the Universities of Messina (Messina, Italy), Catania (Catania, Italy) and Salerno (Salerno, Italy).

For each case, 3 µm-thick sections from two different formalin-fixed paraffin-embedded representative tissue blocks of the primary tumour and metastatic lymph nodes (at least four for each case) were tested for immunohistochemistry (IHC) against HER2. In detail, the immunohistochemical analysis for the evaluation of HER2 status was carried out by using DAKO HercepTest™ kit (Dako, Glostrup, Denmark) with an automated procedure (DAKO Autostainer Link48), according to manufacturer’s instructions. An antigen retrieval pre-treatment was performed by 3 cycles in 0.01 M citrate buffer pH 6.0 in a microwave oven at 750 Watt. Each immunostained section was evaluated by the following score: 0 (absent staining), 1+ (faint and discontinuous membranous staining in <10% of neoplastic elements), 2+ (light to moderate lateral, baso-lateral or complete membranous staining in >10% of neoplastic elements), 3+ (strong, intense lateral, baso-lateral or complete staining in >10% of neoplastic elements). In order to avoid a significant inter-observer variability in HER2 immunohistochemical scoring, after a first assessment in each unit of pathology, all the immunostained slides were reassessed by two observers (AI and GT), who were blinded to the previous paired data, and in a random order. In case of disagreement, cases were jointly discussed by using a double-headed microscope, until agreement was reached.

Fluorescence *in situ* hybridization (FISH) analysis was performed by using HER2 FISH PharmDx™ kit (Dako, Glostrup, Denmark) in those cases showing 2+ and 3+ immunostaining. Gene amplification was recorded when the ratio HER2/centromeric probe for chromosome 17 (CEP17) signal was >2.0.

Fleiss-Cohen weighted k statistics were used to assess the concordance rate between HER2 status of the primary GC and metastatic synchronous lesions. *k* values between 0 and 0.2 were regarded as no agreement, between 0.21 and 0.4 as fair agreement, between 0.41 and 0.6 as moderate agreement, between 0.61 and 0.8 as substantial agreement, and between 0.81 and 1 as almost perfect agreement. The statistical correlations between HER2 status and the other histopathological parameters were investigated by using Chi-squared test. This test was also carried out to assess whether any difference in the clinico-pathological parameters was present between discordant and concordant GC. A *p*-value lower than 0.05 was considered to be statistically significant. Data were analyzed by using the SPSS package version 6.1.3 (SPSS, Chicago, IL, USA).

## 5. Conclusions

In conclusion, our multicenter retrospective analysis confirms that discordance in HER2 status may be present between GC and synchronous metastatic lymph nodes. Although at present the simultaneous determination of HER2 in advanced gastric cancer and matched metastatic lymph nodes is not mandatory, the evidence that the synchronous metastases from GC may have a different HER2 status compared to the primary tumour is of remarkable significance, as it may influence the therapeutic management and impact the prognosis of the patients. As a consequence, the analysis of HER2 status in synchronous metastatic lymph nodes may provide significant additional information for the patients since it may be of help in the identification of possible eligible candidates for Trastuzumab-based therapy, even among patients with HER2 negative primary GC.
